# Editorial: Nutritional approaches in chronic liver diseases

**DOI:** 10.3389/fnut.2024.1480541

**Published:** 2024-09-13

**Authors:** Santiago Rodríguez Villafuerte

**Affiliations:** ^1^Department of Hepatology, Hospital Vozandes, Quito, Ecuador; ^2^Directorate of Postgraduate Studies in Health Sciences, Universidad de las Américas, Quito, Ecuador

**Keywords:** liver disease, nutritional approach, therapeutic, cirrhosis, metabolic dysfunction-associated steatotic liver disease (MASLD)

We are currently at a pivotal moment regarding the awareness, diagnosis, treatment, and management of liver diseases worldwide. The burden of these conditions extends beyond mere epidemiological data—over two million deaths annually are attributed to liver diseases (4% of all global deaths). It is also assessed through composite health indicators that combine two variables: quality and quantity of life. Liver disease has the greatest impact on individuals aged 25 to 49 years, where it ranks as the twelfth leading cause of disability-adjusted life-years (DALYs) (1).

Another approach to evaluating the burden of these conditions is through the investment allocated for research. According to data from the National Institutes of Health, the U.S. government is projected to allocate $954 million in 2024 for the study of liver diseases (2).

Despite regional differences, the global significance of liver diseases is evident (1).

Despite efforts to ensure timely diagnosis and advances in treatment (3), delays and inadequacies in routine clinical care hinder the recognition of macro- and micronutrient deficiencies, resulting from metabolic disorders—an inherent characteristic of chronic liver diseases—thereby preventing adequate nutritional support for these patients (4, 5).

While there are recommendations for using nutrition as an adjunctive treatment in the multidisciplinary management of these patients (6, 7), nutritional interventions are not frequently implemented.

The following sections outline key aspects for managing certain liver diseases. In cirrhosis of any etiology, malnutrition and sarcopenia have been associated with higher mortality and reduced survival. A meta-analysis by Cui et al., indicates a prevalence of 41%, and suggests incorporating sarcopenia screening into the initial evaluation of every cirrhotic patient.

On the other end of the spectrum, metabolic dysfunction-associated steatotic liver disease (MASLD), the most common chronic liver disease and a risk factor for cardiovascular disease and mortality worldwide, has been studied by Liu et al., who propose that timely and appropriate supplementation with vitamins and trace elements may aid in disease recovery or delay its progression, thereby improving patient outcomes.

Similarly, studies by Li et al., and Chen et al., demonstrate that dietary intake of folic acid, choline, vitamin B1, and vitamin B2 may be associated with hepatic steatosis, providing insights into potential dietary strategies for postmenopausal women. Additionally, magnesium supplementation may prevent and treat hepatic steatosis, with variations depending on gender and ethnicity.

In summary, nutritional therapy plays a crucial role in the management of liver disease, and its omission could negatively impact clinical outcomes and the quality of life of affected individuals.
